# The timing of agricultural production in hazard-prone areas to prevent losses at peak-risk periods: A case of Malawi, Madagascar and Mozambique

**DOI:** 10.4102/jamba.v8i2.179

**Published:** 2016-01-13

**Authors:** Leandri Kruger

**Affiliations:** 1 African Centre for Disaster Studies, North-West University, South Africa

## Abstract

Hazard-prone areas in southern Africa experience many natural hazards, which include cyclones, floods and droughts. The severe climatic conditions of southern Africa have an especially large impact on the agricultural practices of small-scale farmers. These hazards should be mitigated to ensure more resilient communities and food security. This study mainly focuses on the timing of agricultural production in hazard-prone areas to prevent losses at peak-risk periods by adapting the agricultural cycle. This study focuses on the agricultural activities of small-scale farmers in Malawi, Madagascar and Mozambique. A literature review is presented, and a mixed-method research design were followed to determine the timing of production followed by these small-scale farmers and its impact on production and food security. Although this study found that the small-scale farmers generally plant with the first rains, it is recommended by literature that early planting should be practised to ensure optimal production. It is also recommended that small-scale farmers should implement water-management techniques for dry periods, and when farmers practice late planting, the use of residual-moisture retention should be utilised as a mitigation measure. This will in effect ensure that the communities are less vulnerable during peak-risk periods by improving or ensuring food security. Therefore, adapting the planting and production time in these hazard-prone areas at peak-risk periods could limit losses and increase communities’ resilience.

## Introduction

Southern Africa is exposed to natural hazards in the context of droughts, cyclones and floods, which puts communities at high risk for disasters. This high risk to disasters is due to the combination of a population’s vulnerability and its exposure to these natural hazards. In this study, vulnerability is directly related to the prevalence of small-scale farmers who are dependent on agriculture and livestock rearing as their primary way of earning a livelihood. The possibility of natural hazards due to climate change might be a threat to vulnerable populations, fragile environments and economies, especially in the poor states of southern Africa. Therefore, there is a need to promote the importance of adaptation strategies and risk-reduction measures to reduce risks through the development of resilience in hazard-prone areas.

In support hereof, the Food and Agricultural Organisation (FAO) has adopted a corporate strategy to provide support and assistance at the local and national level to strengthen and implement policy and capacity to reduce risks and adapt to the impact of climate change. The FAO is focused on building resilience in the context of livelihood systems, which when threatened by severe natural events and hazards might disable and devastate already vulnerable populations.

Rural communities in southern Africa are often the most vulnerable when exposed to natural hazards because their welfare is dependent solely on income from the productivity of livestock and agricultural resources. This research is mainly targeted at livelihood resilience relating to agriculture and food security. The implementation of different intervention activities in order to build resilience has focused on improved methods and techniques to reduce the risk of disasters. The FAO aims to prove that the use of effective agricultural practices can assist in building more resilient communities in the face of the increased exposure to natural hazards.

Therefore, this study aims to determine the contribution of key technical activities to increase the resilience and preparedness of small-scale farmers in Malawi, Madagascar and Mozambique when exposed to natural hazards in southern Africa. This study forms part of a study that was done for the FAO, conducted by the African Centre for Disaster Studies (ACDS) at the North-West University. Although the project report focused on five technical areas, this study will focus on only one of the five technical areas, namely the timing of production in hazard-prone areas to prevent losses at peak-risk periods.

This article will include an in-depth literature review, followed by an explanation of the research methodology used for the study. The results of this study will be given and analysed, and discussions on the findings will aim to make links between the data and the technical area under investigation. Finally, the article will conclude with recommendations on the technical area under investigation.

### Literature review

There are numerous studies which aims to ‘measure’ resilience (Carpenter *et al.* 2001; Tulane University State University of Haiti 2013; Vaitla *et al.* 2012). According to Turnbull, Sterret and Hilleboe (2013), resilience is ‘not a fixed end state, but a dynamic set of conditions and processes’. For the purpose of this report, the term resilience is defined, following Folke *et al.* (2010), as:

The capacity of a system to absorb disturbance and reorganise while undergoing change so as to still retain essentially the same function, structure and feedbacks, and therefore identity, that is, the capacity to change in order to maintain the same identity. (p. 20)

The Food and Agriculture Organization of the United Nations (FAO) (2013) defines resilience as follows:

… the ability to prevent disasters and crises as well as to anticipate, absorb, accommodate or recover from them in a timely, efficient and sustainable manner. This includes protecting, restoring and improving livelihoods systems in the face of threats that impact agriculture, nutrition, food security and food safety. (p. 9)

In order to measure resilience, one needs to relate it to real-world variables and indicators. Therefore, this paper will aim to determine to what extent the selected agricultural and food-security activities contribute to the ability of small-scale farming to absorb natural hazards and risks to essentially remain productive while undergoing change.

The occurrence of extreme weather events such as drought, floods and cyclones are expected to increase in future, mainly due to the impact of global climate change (Ding, Schoengold & Tadesse 2009:28; McDonald 2013:1). Climate change is one of the greatest environmental, social and economic threats facing the planet today and threatens to affect food and water resources that are critical for livelihood in sub-Saharan Africa, especially for those communities which rely solely on rain-fed agriculture for their livelihood (Zhou *et al.* 2010:3). According to the FAO (2013:8), small-scale farmers represent 90% of the rural poor and make up the majority of the world’s hungry population. The FAO also argues that the underlying risk of food and nutrition security could be addressed by the application of prevention and mitigation measures in approaches to farming (FAO 2013:8).

According to Van Zyl (2008), drought is a major climate feature in southern Africa that has a devastating impact on agricultural activities. Drought is a climatic condition of extreme dryness that is so severe that it reduces soil moisture and water levels to below the minimum requirements that are necessary for crops, animals and economic and social systems. The effect of drought is more severe in developing countries, which depend on dry-land farming, and a large part of sub-Saharan Africa is susceptible to drought and frequently experience droughts (Mulugeta *et al.* 2007:6).

Similarly, Van Zyl (2008:34–37) notes that atmospheric extremes are the most common causes of floods and flash floods. These floods can vary from tropical cyclones, which are associated with lengthy rainfall periods over a large drainage basin, to convectional storms over small basins that can occur randomly. Semi-predictable seasonal rains, the saturation of low hydrological basins and the confluence of rivers are also likely to increase the annual wet-season floods in tropical areas. Floods and flash floods may not only cause loss of life, but it may also cause damage to properties and increase the spread of diseases. For these reasons, defences against floods are essential to protect communities (Mulugeta *et al.* 2007:5–6). The hazard profile of southern Africa thus has a significant impact on the primary economic activities of the region, which remains based on agriculture.

### Changing the timing of crop production

One of the most important factors that affect agricultural productivity in southern Africa is the high spatial and temporal rainfall variability. This is reflected by the dry spells and periodical droughts and floods that these areas experience. For this reason, the altering of production timing will be explored to prevent severe agricultural losses at peak-risk periods in hazard-prone areas.

In southern Africa, the peak of hazards for droughts are likely to occur at random intervals whilst floods and cyclones are more likely to occur in the period between December and March. This directly influences the agricultural rain-fed season and production, especially in areas prone to hazards. The timing of crop production in these hazard-prone areas of southern Africa can be altered slightly to reduce or prevent the climatic impact at peak-risk periods and to ensure that small-scale farmers and their production period are more resilient during peak-risk periods by either early or late planting of crops.

#### Early planting

Early planting is when crops are planted earlier in the planting season to ensure that they will be mature enough at peak-hazard periods. Early planting might significantly increase dry-matter accumulation and improve crop yields in comparison with normal planting times (Bannayan, Rezaei & Hoogenboom 2013:57). However, Laux *et al.* (2010:1259) also explain that planting crops too early might lead to crop failure. Therefore, early planting is when crops are being planted with the first rains, which increases the risk of crop failure.

#### Late planting

Late planting is when crops are planted after the normal planting season or when the planting season is delayed until the severe hazards have passed. Thus, late planting is considered to be the planting of crops after the rainy season. Laux *et al.* (2010:1259) state that, by planting crops too late in a season, one might reduce the crops’ valuable growing time and thus yield whilst it can also delay or reduce crops’ initiation and maturity (Buddhaboon, Jintrawet & Hoogenboom 2011:270). According to Bannayan *et al.* (2013:62), the late planting of crops holds a few advantages and disadvantages for crop production. The advantages include that the stress of drought during the early growth stages could be avoided because more water is available. The disadvantages include that the crops could be exposed to higher temperatures while maturing and the risk of scarcity of rain in the final stages of crop growth, except if late planting is combined with short-cycle varieties and with good cropping techniques. In the following section, the adaptation of crop production will be investigated to determine whether these adaptations influence farmers’ resilience.

#### The adaptation of the production season to increase the resilience of small-scale farmers

Laux *et al.* (2010:1260) reflect that the planting date of crops are of central importance for agricultural productivity. It is therefore critical for the agricultural sector, especially small-scale farmers, to increase their resilience to hazards by adapting their production season. A key component in adapting agriculture, identified by Howden *et al.* (2007:19693), is to change the agricultural practices of farmers at the management level. The existing adaptation options at the management level are basically an extension of existing production-enhancement activities in response to the risk entailed in potential climate change. Various management-adaptation options are identified by Howden *et al.* (2007:19693) to cope with the projected climatic hazards, but a few are focused specifically on the purpose of this study:

- altering the timing as well as the location of farmers’ cropping activities
- annual climate forecasting used to reduce the agricultural production risk
- adapting irrigation timing and water management in crop fields
- a wider use of technologies to ‘harvest’ water and to conserve soil moisture.

By combining some of these adaptations, negative impact could be limited and positive impact could be increased. According to Sacks *et al.* (2010:607), farmers around the globe are going to face a very challenging task because a growing population demands an increase in food production. One of the most important strategies that farmers could use in the face of a changing climate in order to maintain or even increase their crop yields, especially in developing countries, is to adjust their planting dates. The quantity and quality of crop production can be changed significantly by as small a factor as the appropriate selection of planting dates. By changing the planting date of crops, one is raising the stakes. On the one hand, you can increase crop production and reduce the risk hazards having an impact on crops, but on the other hand, crops can be exposed to severe environmental conditions (Bannayan *et al.* 2013:57).

## Research methodology

The research methodology that was followed for this study will be discussed in detail in this section. A thorough literature review was conducted and a mixed-methods research approach was applied.

### Literature review

An in-depth literature review was conducted for this study to provide a theoretical overview of disaster risk reduction and resilience interventions in the agricultural sector. The literature review focused mainly on the timing of production in hazard-prone areas to prevent losses at peak-risk periods.

### Empirical study

For this study, a mixed-method research design was used. According to De Vos *et al.* (2013:443), a mixed-methods research approach is adopted when ‘one data set provides a supportive, secondary role in a study based primarily on the other data type’. In this study, quantitative data was gathered through surveys, and qualitative data was obtained from focus group and face-to-face interviews. The combination of these two research designs gave the researcher the ability to draw on multiple types of data and results to structure arguments for the conclusion of this study.

#### Sampling

The survey sample was targeted at the beneficiaries of various agricultural and food-security activities undertaken by FAO and partners over the past five years under Disaster Preparedness ECHO (DIPECHO) funding. The research was conducted in Malawi, Madagascar and Mozambique. A combined total of 1110 respondents were interviewed in this study. Madagascar represented 30.4% (*N* = 337), Malawi 29.4% (*N* = 326) and Mozambique 40.3% (*N* = 447) of the participants. The beneficiaries that were interviewed were identified by FAO partners in the various countries, and the respondents were then randomly selected from these target populations by enumerators, and the survey was completed.

#### Questionnaire design

The survey that was used in this study was designed to allow the questions to be analysed in various ways. To evaluate the contribution of each of the FAO’s activities, Likert-scale items as well as questions which would allow the researchers to measure disaster resilience were included, based on the theoretical foundation as discussed previously. The literature review was conducted to identify a framework which could be used to measure disaster resilience. It was decided by the researchers to use the Department for International Development’s (DFID) Sustainable Livelihoods Framework (SLF) because it gives a more holistic picture of the dimensions of resilience. This framework is also better suited to the objectives of the FAO’s activities. According to this framework, resilience has the following dimensions (called ‘capitals’): natural, social, human, economic and financial. These capital domains were used to structure the research tool. Questions were therefore included in the survey to test the various dimensions of the SLF. The draft survey was sent to the FAO and the in-country teams for their comments.

To test and validate the research tools, a workshop was held in Malawi. The aim of the workshop was also to train key personnel of each country team (Malawi, Mozambique and Madagascar) on the utilisation of the research tools, technology and techniques. To this end, during the workshop, a pilot study was carried out in the Chikhwana district in Malawi to see if there were any unforeseeable problems with the application of the questionnaire and to test the research technology in a live environment. During the pilot study, the quantitative questionnaire was changed in line with the learning of the pilot team in the field. After the workshop and pilot study, the final questionnaire was compiled and agreed to by FAO. The focus-group questionnaire was designed to elicit supplementary information which could be used to support or illuminate some findings from the survey data. The focus-group questionnaire was also sent to the FAO and the in-country teams for their comments before it was finalised.

#### Data gathering

The nature and extent of the research allowed the research team to explore new methods of multi-site, multi-language, data collection by making use of electronic tablets and offline data capturing. The research team decided to use proprietary server-side software (called Survey Analytics) which allows for multi-language support and offline capturing of data with synchronisation capabilities. The final questionnaire was translated into Portuguese and French. Although the questionnaire was thus administered in three languages, the software allowed for the writing of the data to one central database. In doing so, the research team could constantly throughout the field research phase track data entries, research progress, identify problems with the answering of certain questions and make live updates to the questionnaire which was synced with all the field electronic tables respectively. This data-gathering technique and lessons learned were in itself found to be extremely valuable, especially for an organisation such as FAO who are constantly relying on accurate and timely data from remote sites.

In each country, the questionnaires were completed by trained enumerators, and the interviews took place in the respective target areas. From the populated database, the primary data was extracted for analysis. In addition to survey data, ten participatory focus-group discussions were held in each country. These discussions were recorded, transcribed, coded and analysed.

#### Data analysis

For the quantitative data analysis, each section was reported on per statement as a percentage. Qualitative data obtained through focus-group discussions were analysed by means of conceptual (thematic) analysis.

## Findings

### Demographical information

In this section, a demographical overview are given of the survey areas in the countries studied. More specifically, this section provides the information pertaining to the planting and harvesting times and the occurrence of natural hazards.

#### Survey area

**FIGURE 1:**
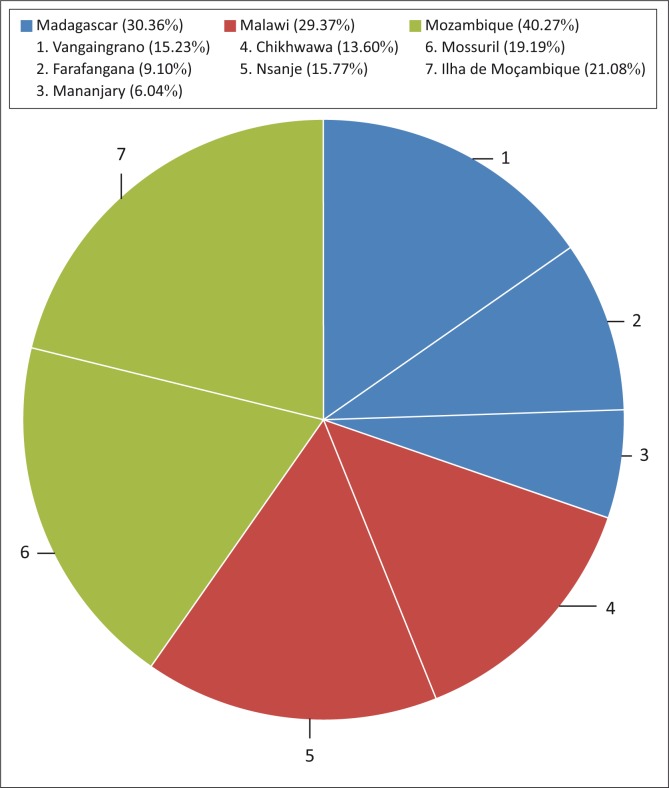
Survey area.

The research was conducted in Malawi, Madagascar and Mozambique. A combined total of 1110 respondents were interviewed. Mozambique represented 40.3% (*N* = 447) of participants, Madagascar 30.4% (*N* = 337) and Malawi 29.4% (*N* = 326) (Figure 1). The areas or districts surveyed as well as their representation within the different countries are provided in Figure 1.

#### Gender representation

Both male and female respondents were interviewed. Both genders contributed equally across the countries with females accounting for 47.75% and males for 52.25% of respondents.

### Natural hazards and their occurrence

**FIGURE 2:**
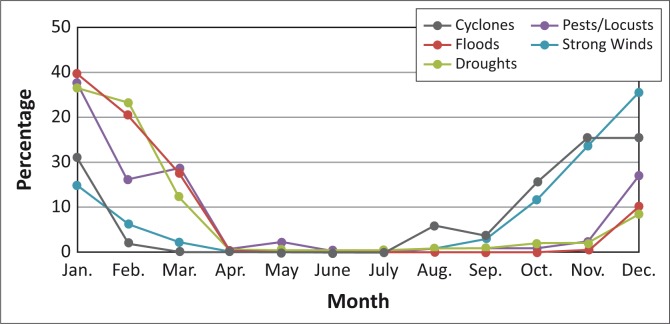
Occurrence of hazards likely to occur in Malawi according to month.

**FIGURE 3:**
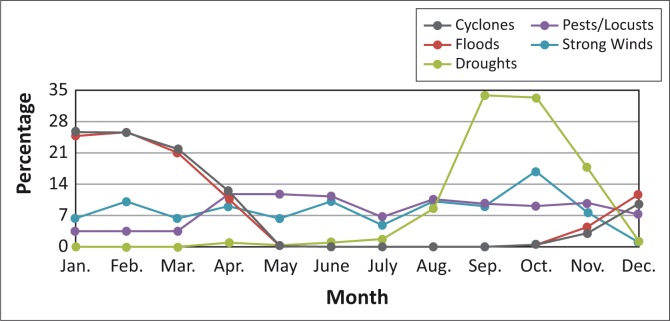
Occurrence of hazards likely to occur in Madagascar according to month.

**FIGURE 4:**
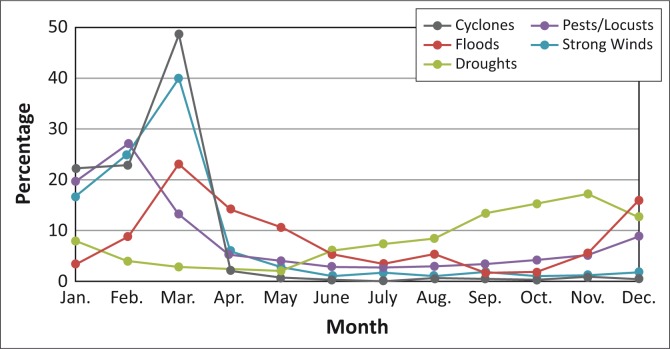
Occurrence of hazards likely to occur in Mozambique according to month.

Figure 2, Figure 3 and Figure 4 provide the occurrence of hazards according to the months in which they are likely to occur in the three countries.

From Figure 2, it is evident that the majority of respondents from Malawi indicated that strong winds tend to occur during the time period October to January. Floods and droughts tend to occur mostly during the months of January and February. They are confronted with pests or locusts mostly during the time period December – March.

The majority of respondents from Madagascar indicated that cyclones and floods tend to occur during the time period January to March. Droughts are mostly experienced during the months of September and October. Pests and locusts are experienced from April to December. It is also evident from the figure that, according to the respondents, it is not rare to find strong winds in Madagascar throughout the year (Figure 3).

The majority of respondents from Mozambique indicated that cyclones tend to occur during the time period January –March with the highest occurrence in March. Floods are experienced mostly in the months of December – March. Droughts tend to occur mostly from September to December. Problems with pests and locusts are mostly experienced from January to March. Strong winds are mostly experienced in March and also tend to occur in January and February (Figure 4).

#### Planting and harvesting time

Table 1 and Table 2 provide the percentage of respondents who indicated their planting and harvesting times for the various crops per country, respectively. The percentages presented in bold letters in Table 1 and 2 indicate the period of the planting and harvesting times of the different crops in the respective countries. It is important to note that the ability to irrigate maize crops enables Malawian respondents to plant maize outside of the typical planting time of November – January. The aforementioned enables Malawian producers to harvest irrigated maize from June to October.

**TABLE 1:** Planting times (percentage of respondents).

Crop	Jan.	Feb.	Mar.	Apr.	May	June	July	Aug.	Sept.	Oct.	Nov.	Dec.
**Malawi**												
Maize (irrigated)	-	1	**14**	**21**	**23**	**20**	**11**	6	3	2	-	1
Maize (rain fed)	6	-	-	-	-	-	-	-	-	-	**18**	**75**
Sorghum	4	1	-	-	-	-	-	-	-	-	**21**	**73**
Cotton	**10**	1	-	-	-	-	-	-	-	1	**16**	**74**
Rice (summer)	**25**	6	-	-	2	2		2	-	3	**17**	**45**
Rice (winter)	**17**	11	-	6	-	**17**	**11**	6		-	**11**	**22**
Millet	3	2	-	-	-	-	-	-	-	1	**26**	**67**
**Mozambique**												
Maize (irrigated)	**45**	**38**	2	-	-	-	-	2	4	-	2	8
Maize (rain fed)	**22**	2	-	-	-	-	-	-	-	-	**11**	**65**
Rice (summer)	**16**	**34**	-	-	-	-	3	6	-	-	6	**34**
Rice (winter)	**33**	**20**	7	7	-	-	-	-	-	7	7	**20**
Cassava	**11**	2	1	1	-	-	-	2	**8**	**11**	**18**	**47**
**Madagascar**												
Rice (summer)	6	-	1	1	1	1	-	2	2	**15**	**19**	**52**
Rice (winter)	1	-	3	**13**	**18**	**19**	**11**	**20**	8	4	1	3
Cassava	-	-	1	2	1	2	**14**	**51**	**10**	**9**	3	6
Sweet potatoes	-	1	**44**	**30**	3	5	5	7	0	2	2	-

The percentages presented in bold letters indicate the period of the planting and harvesting times of the different crops in the respective countries.

**TABLE 2:** Harvesting times (percentage of respondents).

Crop	Jan.	Feb.	Mar.	Apr.	May	June	July	Aug.	Sept.	Oct.	Nov.	Dec.
**Malawi**												
Maize (irrigated)	-	-	1	1	1	**7**	**17**	**33**	**22**	**11**	5	2
Maize (rain fed)	1	4	**56**	**37**	1	-	-	-	-	-	-	-
Sorghum	2	**13**	**41**	**30**	**10**	3	1	-	-	-	-	-
Cotton	-	-	8	**24**	**32**	**27**	8	-	-	-	-	-
Rice (summer)	-	2	**12**	**17**	**30**	**28**	7	2	-	2	2	-
Rice (winter)	-	-	**19**	**13**	**6**	**25**	6	-	**6**	**6**	**19**	-
Millet	6	**40**	**40**	**9**	3	-	-	-	-	-	-	-
**Mozambique**												
Maize (irrigated)	2	-	4	**38**	**19**	**29**	2	2	2	2	-	-
Maize (rain fed)	-	1	**10**	**37**	**19**	**21**	**10**	1	1	-	-	1
Rice (summer)	3	-	**10**	**20**	**20**	**37**	**7**	3	-	-	-	-
Rice (winter)	-	-	**14**	**21**	**43**	**21**	-	-	-	-	-	-
Cassava	-	-	2	6	4	9	6	**18**	**31**	**18**	6	2
**Madagascar**												
Rice (summer)	2	2	3	6	**70**	**11**	2	-	1	-	1	3
Rice (winter)	9	3	1	1	4	2	4	6	2	**11**	**23**	**34**
Cassava	2	4	5	**12**	6	4	**20**	**36**	3	2	2	3
Sweet potatoes	-	2	2	3	-	8	**14**	**14**	**36**	**10**	3	6

The percentages presented in bold letters indicate the period of the planting and harvesting times of the different crops in the respective countries.

**FIGURE 5:**
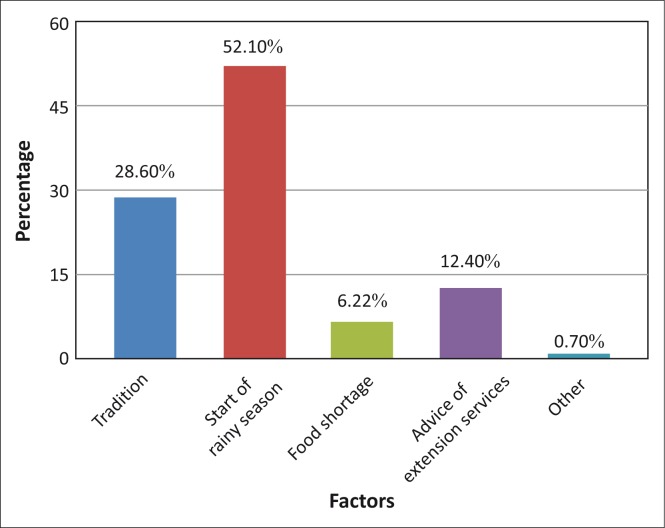
Factors determining planting time.

The first question proposed to respondents was used to indicate which factor(s) determine(s) their planting time. As can be seen from Figure 5, more than half of the respondents (52.10%) indicated that the start of the rainy season determines their planting time. Comparing the results from the different countries, it was found that the respondents from Mozambique and Malawi mainly start planting when the rainy season starts. Although 44.26% of Madagascar’s respondents indicated that their planting season is traditional (April – December depending on the crops being planted), 25.85% still indicated that they start their planting season when the rainy season starts.

**FIGURE 6:**
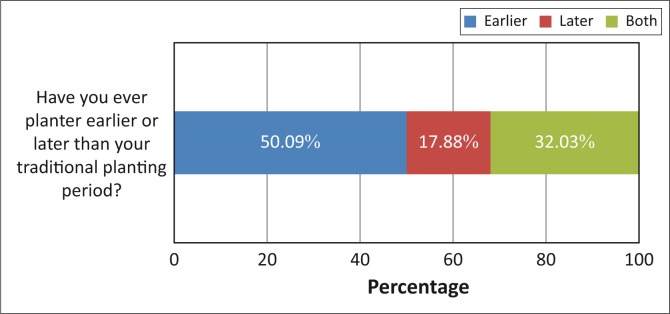
Planting earlier or later than the traditional planting period.

Respondents were asked whether they ever planted outside of the traditional planting time (Figure 6). In response, 50.09% of the respondents indicated that they practice early planting whilst 17.8% of the respondents indicated that they practice late planting.

**FIGURE 7:**
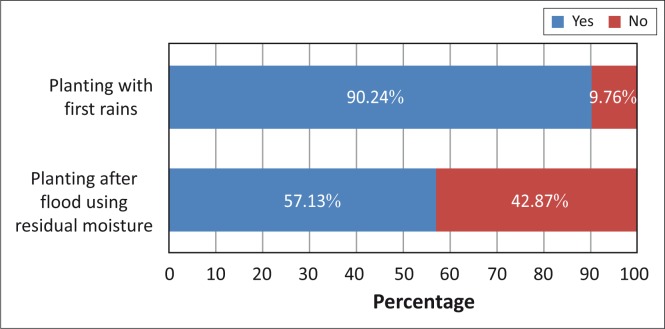
The timing of planting.

Respondents were asked whether or not they plant with the first rains or after a flood with residual moisture (Figure 7). A combined majority of the respondents (90.24%) indicated that they plant with the first rains whilst 57.13% indicated that they plant after the floods, utilising residual moisture. The results from different countries support these findings as the majority of the respondents from all the countries agreed that they practice both planting with the first rains and using residual moisture after floods. They further indicated that both of these techniques were very useful for their planting season.

**FIGURE 8:**
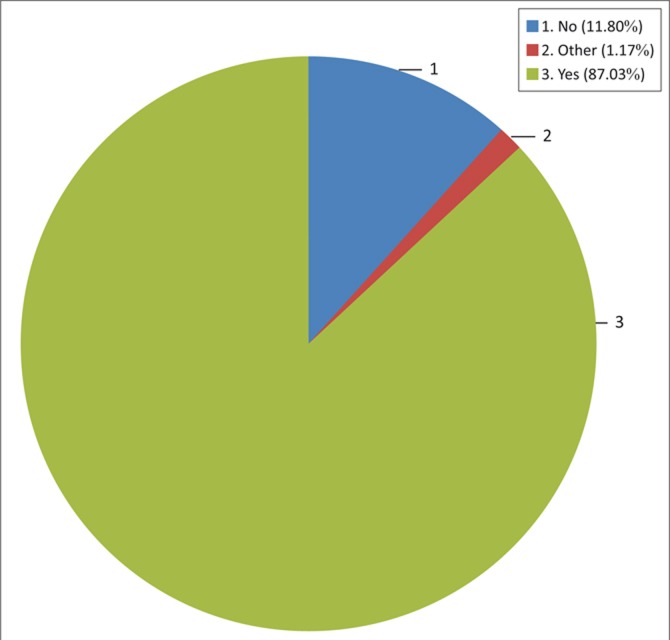
The perceived usefulness of planting with the first rains.

**FIGURE 9:**
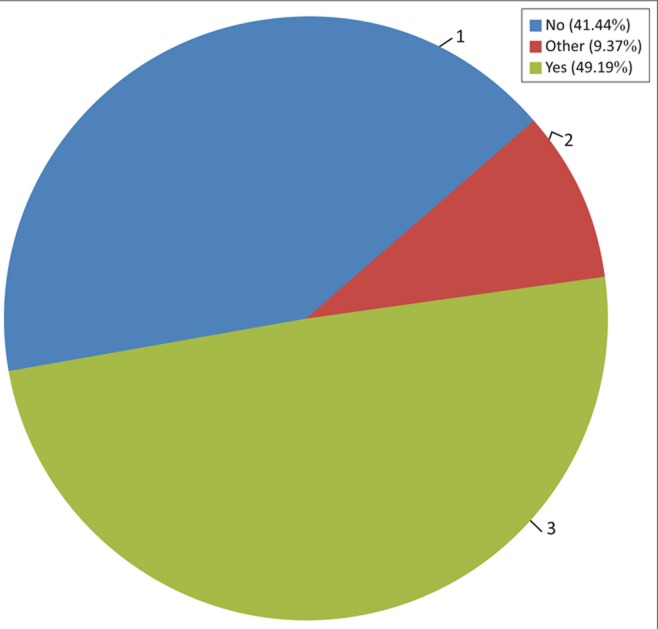
The perceived usefulness of planting after floods, using residual moisture.

Figure 8 and Figure 9 present the respondents’ view of the usefulness of the techniques applied. In Figure 8, data show that 87% of respondents agreed that planting with first rains is useful whilst the data in Figure 9 show that 41.19% indicated that they see planting in residual moisture, following floods, as useful.

The countries included in the survey differ in terms of, amongst others, their respective resource base, environmental conditions and production practices. Because the findings in this section support claims from this survey, each country’s timing of their production period will be discussed separately in the following section.

### Madagascar

The climatic information given earlier indicates that Madagascar experiences cyclones and floods during January to March, droughts from September to October, pests directly after the flood period from April to December and strong winds throughout the year. In the above survey results, it was indicated that the main planting time in Madagascar is from March to September. These results correlate with each other, showing that the planting season for respondents from Madagascar is primarily during their traditional season (44.26%) and that they also plant at the start of the rainy season (25.85%). In the focus-group interviews, most of the households indicated that they have planted their crops prior to the traditional planting season, but very few of them (24.32%) planted after the traditional season. The reason for this is that the period after the traditional planting season is a rainy season that coincides with the spreading of pests like rats and birds that destroy crops. Planting outside of the traditional planting period is a risky endeavour due to the threat of cyclones and floods, and the climate is also not suitable for certain cultivars. In areas like Sandra in Madagascar, the planting period for farmers differs. Due to water shortages in this area, farmers are dependent on the first rains. In this area, there are no dams or other water-storage facilities to store water prior to the rainy season. In addition, floods occur during the rainy season which makes it unsuitable for planting rice. In fact, their crops are destroyed by the floods every year, and currently, no mitigation measures are in place to address this matter.

Malagasy respondents indicated that they practice early planting prior to the traditional planting season (33.63%). For them, early planting holds certain advantages when using short-cycle crop varieties that reach their maturity prior to the occurrence of hazards like cyclones and floods.

Even though the farmers in Madagascar prefer not to practice late planting because of the risks that it involve, 60.83% of them agreed that planting after floods to use residual moisture is a very useful technique. In the focus-group interviews, respondents indicated that utilising residual moisture is an efficient technique. Debris that is left from floods can also be used as mulch and/or fertiliser. The late planting of crops can be beneficial because the land is more fertile, resulting in more efficient production.

Food security is a major issue for the farmers of Madagascar. To ensure their food security, they plant yams and cassava during dry periods, which is earlier than the traditional planting period, as these crops are more resistant to drought. However, in order to increase their production, the majority of farmers use traditional farming techniques and wait for the first rains to start planting their crops. During the traditional planting season, they make use of short-cycle varieties, which ensure that the fields can be maintained before the next planting time and also ensure a better harvest. From the focus-group discussions, it is evident that farmers in Madagascar are adopting both early and late-planting techniques. By using these techniques combined with the use of short-cycle varieties, their exposure to severe hazards is reduced and their crop production increased. This makes the farmers more resilient. Thus, to ensure that the farmers of Madagascar’s resilience is enhanced and also to ensure and improve food security, producers are managing their planting calendar to avoid hazard-prone times.

### Malawi

In Malawi, 10 focus-group discussions were held with groups of farmers. The climatic data indicated that Malawi experiences heavy rainfall as a result of cyclone activity during the months of October – January and floods in January – March. Strong winds are experienced from November to January. It was also indicated by the respondents that they plant from March to July. The respondents indicated that they practice both early and late planting. Some farmers indicated that they use traditional planting time where they dry plant, having observed the problematic weather patterns. In total, 54.91% of the respondents indicated that they make use of early planting, and 98.46% indicated that they plant with the first rains.

Many of these respondents in Malawi plant early or, as they would say, as soon as possible after the first rains have fallen. The respondents indicated that, through early planting, they are able to harvest more and better-quality products because the crops use all the moisture in the soil, and they are able to avoid damage from pests and diseases. According to the respondents, the other advantage of early planting is that, because of this planting period, they utilise the good rainfall and farmers are able to plant twice during this time period. By early planting, crops also escape dry spells and droughts, and when the floods come, the crops are already mature, and farmers are still able to harvest some of their crops. Early planting increases production, and the crops are exposed to fewer hazards, which enable these respondents to be more resilient against hazards and disasters.

The Malawian respondents mentioned that they also practise late planting. The quantitative results indicated that 54.91% of respondents practice late planting. Although they indicated that this time period (late planting) could be beneficial, its disadvantages and risks are too high, which is why the percentage found in the quantitative results is significantly lower. The disadvantages of late planting are that there is an increased risk of crops being invaded and damaged by pests. Secondly, farmers also find this period to be very unpredictable because, even though the floods have passed, they can still not be certain as to whether or not more floods are on their way. Late planting also leads to poor seed germination, which causes reduced crop production. Therefore, data from the respondents in Malawi indicate that early planting and planting with the first rains are effective: It not only increases their production, but it also makes them less vulnerable and more resilient against hazards and disasters, in contrast to late planting.

### Mozambique

The two districts surveyed in Mozambique experience subequatorial climates. In the dry months, these districts experience a humid tropical climate with winds. The farmers can experience an annual precipitation of approximately 800 mm, peaking at 100 mm – 150 mm per month in the rainy season. After analysing the data on the average rainfall in Mozambique between 2009 and 2013, it is evident that the rainfall is erratic. These two districts are also characterised by sand-rich soil and open-clay soil. The communities that are situated next to the coastal area in these districts experience a lower crop production due to acidic sand-rich soil with little water retention. In contrast, inland communities experience better crop harvests, partly due to less acidic open-clay soils. The climatic information indicates that Mozambique experiences cyclones from January to March, floods from December to March, droughts from September to December and pests and strong winds during the same period as the cyclones and floods. Here, the respondents also indicated that they plant from November to February.

During the focus-group interviews, the respondents indicated that early planting is the best strategy for them to follow to gain the best cropping yield. These results from the focus-group interviews correlate with the quantitative results where it was found that 58.92% of respondents practice early planting and 95.71% plant with the first rains. According to the respondents, early planting ensures faster-growing crops because the germination of the crops occur before the arrival of diseases and pests and the crops are stronger and more resistant. However, some of the farmers also indicated that early planting hold very high risks because, if the rains come later than predicted, the seeds that they have planted may die. In order to avoid this risk, farmers use only half of their seed after the first rains and the rest with the second rains. Therefore, the respondents from Mozambique suggest that planting after the second rainfall is the safest planting time in spite of weaker and more vulnerable production.

In these two districts in Mozambique, it was found that planting before the first rainfall is a strategy that can increase production and, subsequently, income. In addition, it contributes to the resilience of crops regarding droughts, pests and strong winds. It was further found that planting with the second rainfall, which is their traditional planting time, is safer although it does not minimise their crops losses if a hazard or disaster affects their district. Late planting (after their traditional planting time) leads to even greater losses, which is not an option for them at all.

The only crop that is an exception regarding planting time is cassava because cassava is less affected by drought in comparison to other crops. There is no specific planting time for this crop, and it could be planted at any time during the year, depending on the household’s needs. This crop ensures that communities are less vulnerable during disasters and contributes to their resilience to disaster risk, ensuring these communities’ food security.

## Conclusion and recommendations

The severe hazards that have an impact on agricultural activities in hazard-prone areas in southern Africa should be mitigated in such a way that communities are less vulnerable and more resilient against the impact of hazards and have increased food security. Through a thorough literature review and a mixed-method research design, it was found that intervention regarding the timing of the production and planting of crops was needed to ensure more resilient agricultural activities and less vulnerable small-scale farmers. This study found that the respondents from the countries investigated mainly plant with the second rains. This, however, did not increase their resilience to hazards or make them less vulnerable.

It is suggested that small-scale farmers should practice early planting (planting with the first rains) to ensure an increased resilience at peak-risk periods and to improve their food insecurity. Small-scale farmers should also implement water-management techniques for dry periods, and when farmers practice late planting, the use of residual moisture retention should be utilised as a mitigation measure. Through these recommendations, small-scale farmers will be more food secure and more resilient during peak-risk periods.

Natural hazards have increased in frequency and intensity over the last few decades and are having an increasingly negative impact on the livelihood and food security of rural small-scale farmers. Certain agricultural adaptations are needed in order for these farmers to be able to survive these changing climatic conditions in hazard-prone areas. Building resilience requires a multi-sectoral response, and as such, the FAO addresses resilience as it relates to livelihoods – specifically agriculture and food security and their related activities. Rural communities are amongst the most vulnerable to natural disasters as their livelihoods depend on agricultural production. Whilst one cannot change the fact that hazard-prone areas will continue to be exposed to natural hazards, the impact of these hazards on agriculture and food security can be reduced if the right agricultural practices are implemented.
